# Womens’ Career Progression in an Australian Regional University

**DOI:** 10.3389/fsoc.2021.742287

**Published:** 2021-11-08

**Authors:** Kate White, Anitra Goriss-Hunter

**Affiliations:** School of Education, Federation University Australia, Ballarat, VIC, Australia

**Keywords:** gender, higher education, terms of employment, career progression, flexible work arrangements

## Abstract

This article examines the link between terms of employment (full time, part time and casual) at an Australian regional university and women’s career progression. The literature identifies lack of transparency in recruitment, promotion and retention; mobility and location; and management perceptions of women’s choice to work flexibly as factors impacting on career progression. However, the voices of women working in regional universities and particularly those of professional staff are often not present in current research. This study moves towards addressing this research deficit. Feminist institutionalism is used to analyse the relationship between national legislation, university policies and informal institutional practices in relation to women’s career progression In early 2020, twenty-one women provided written responses to questions on the link between terms of employment and career progression. The main findings tend to support other research about women working in universities; that is, carers need flexible work arrangements. But there are particular differences for women in regional universities who have to travel between dispersed campuses, which brings an added dimension of complexity to career progression. Their choices about terms of employment and fulfilling carer responsibilities resulted in insecure employment for some participants which had an impact on wellbeing and confidence. In addition, care/household responsibilities and the choice to work flexibly had a negative effect on career progression, and managers did not necessarily support flexible work options (despite national legislation that enables employees with child care responsibilities to negotiate flexible work arrangements with managers, and institutional gender equality policies).

## Introduction

Aiming to address the gap in current literature about the experience of women working in Australian regional universities, especially professional staff, this article examines the terms of their employment (full time, part time and casual) and how this might connect with/impact on career pathways and advancement. The research question, therefore, is how might terms of employment affect the working lives, career aspirations, and career progression of women in regional universities.

Twenty-one women participated in the study in February 2020, and were almost equally divided between academic and professional (administrative) staff.

The regional university sector comprises seven universities based in regional Australia; that is, in regional cities rather than metropolitan areas. They have formed the Regional University Network (RUN). Even though regional universities are often central to rapidly growing areas outside metropolitan locations that have witnessed the largest net inflow of population in the past 2 years (ABS, 2021), there was a gap in the literature on women in regional universities that we aimed to start addressing with our research and thus help to contribute to the growth of regions (RUN, 2021). Regional universities attract and retain diverse cohorts–including first-in-family (FiF) and regional and rural students and staff. Goriss-Hunter and Burke (2015), p. 112 note that there are “interconnections between the regional university, a diverse student population, and the local community”.

Like other regional universities, this university is located in a region outside a metropolitan area, has dispersed campuses and is strongly connected to the local community and economy. So, it provided a good case study for our reseach. Women comprise over 70% of professional staff and just over 50% of academic staff (Universities Australia UA, 2020) of the 1,000 plus workforce. Professional staff, sometimes called administrative staff, are defined as those staff not employed to undertake academic work.

## Literature Review

Various barriers influence women’s career progression in higher education (HE). One is the continuing lack of transparency in recruitment, promotion and retention (van den Brink, 2009; Morley, 2014; Acker, 2014), despite universities having equality and diversity policies (Fitzgerald and Wilkinson, 2010). Interventions such as the UK’s Athena SWAN (AS) place university equality and diversity policies under the microscope and would hopefully produce better outcomes for women (Rosser et al., 2019). The Athena SWAN Charter was established in 2005 to address the unequal representation of women and to encourage and recognize commitment to advancing the careers of women in science, technology, engineering, mathematics and medicine (Barnard, 2017). However, Athena SWAN does not foster a bottom-up approach to gender equality (O’Mullane, 2020) and hence the reforms required to facilitate more women moving into leadership. Rather, as Barnard (2017) found, the old masculinist culture remained, with a focus on “fixing” early career women and therefore the commitment to gender equality was more difficult to identify.

Mobility and location can also act as barriers. Limited geographic mobility (González Ramos and Vergés, 2012; White, 2014) can adversely impact on women’s careers. Zippel (2017) demonstrated how women academics can build international networks and be taken more seriously as researchers internationally than at home. Thus, being a woman and a foreigner in another country can be a positive combination rather than ‘an accumulation of disadvantages’ (Zippel, 2017, p. 26). But for some women in regional universities even relocating to another area in Australia can be a challenge because of their responsibility for children and/or elder care (Thomas et al., 2019; Manyweathers et al., 2020).

In addition, the issue of equity policies and practices in relation to women taking up flexible working conditions can be a barrier. It should be noted that the university in this study is committed to providing “reasonable adjustments/flexible working arrangements to the learning and working environment as required, and will use inclusive practices wherever practicable, to ensure that all people have equal opportunity to access and participate in University activities”. It is also obliged to implement national legislation (the Fair Work Act) that enables employees (other than a casual employee) who have worked with the same employer for at least 12 months to request flexible working arrangements if they: are the parent, or have responsibility for the care, of a child who is school aged or younger; are a carer (under the Carer Recognition Act, 2010); have a disability; are 55 or older; are experiencing family or domestic violence, or provide care or support to a member of their household or immediate family who requires care and support because of family or domestic violence. Casuals can request flexible working arrangements if they have been working for an employer for at least 12 months, but they are not entitled to paid days off or notice of termination (Fair Work Ombudsman Australia, 2021).

Early qualitative research suggested that flexible work was critical to women juggling career and family responsibilities and the only way to reconcile work and family (Lewis et al., 2008). While it did alleviate work/life conflict, it often made women who chose flexible work the target of conventional thinking about women being primary carers whose career progression did not need to be supported. Hence, they remained in lower paid work where flexible working hours were acceptable, sadly trading their ambition for this flexibility (White and Burkinshaw, 2019). Most women are keen to progress if only flexible working at senior levels was an option (see White, 2017; Matthews 2019). However, Padevic et al. (2019) found that flexible work options alone would ‘not dismantle the culture of overwork, nor will they dislodge the deep-rooted … association of women with family and men with work” (p. 43). Meekes (2021), p. 5 argues that government and employers could encourage men to share childcare responsibilities by “increasing men’s access to parental leave or by prioritising flexible work arrangements for men”. Targeting policy on job flexibility in Australia, he asserts, could further close the gender gaps in employment and income. Unless there are provisions in policy for men to take up flexible options and encouragement to do so, perceptions of effective career progression will not change. However, “taking parental leave does not have the same consequences for the career progression of mothers and fathers” according to Le Feuvre (2015), p. 39 who points out that even in countries with generous support for working parents “men tend to reap a “paternity bonus” in terms of career progression, while women continue to pay a “motherhood penalty”“. Therefore, the decision to work flexibly can produce unequal outcomes, change career trajectories and push women into career cul-de-sacs (see also Barrett and Barrett, 2011).

Thus, women working in universities can experience multiple forms of disadvantage which, not surprisingly, lead to increased stress (Morrish, 2019) and become evident throughout their careers (Pyke, 2013; Kefting, 2003) as they are blocked for promotion (van den Brink 2009; Kandiko Howson et al., 2018), passed over for higher duties, pushed side-ways (White, 2013) and not acknowledged for their contribution to the work team (Bevan and Gatrell, 2017). Understandably, some women may experience self-doubt and/or resistance to their current working environment (Blackmore and Sachs, 2007; Morley and Crossouard, 2016) and eventually find themselves positioned as outsiders on the inside (Gherardi, 1995).

A good deal has been written about women’s academic career progression in Australia (see for example, Currie et al., 2002; Chesterman et al., 2003; Winchester et al., 2006; Blackmore and Sachs, 2007; Fitzgerald and Wilkinson, 2010; Marchant and Wallace, 2013; Lipton, 2017; Sharafizad et al., 2018), including analysis of why affirmative action initiatives have had minimal impact despite considerable investment over the past 30 years (Fitzgerald and Wilkinson, 2010; Diezmann and Grieshaber, 2019). But there is little research on the careers of professional women in HE. Wallace and Marchant (2011) found that women professional (administrative) managers experienced long hours and presenteeism and needed to adopt a style that privileged a conventional masculine approach to management in order to succeed, while Lawless’s (2017) case study of a junior female professional staff member indicated that sexism, gendered roles and silencing reduced her agency. Meanwhile, Bailey et al., 2017 examined the impact of part-time work on career advancement, and Gander (2017) identified a mismatch between career and promotion aspirations, and opportunities provided by the institution. Other research identified a “them and us” divide between professional and academic staff (Graham and Regan, 2016), and misunderstanding or misrepresentation of the scope of the work and decision-making authority of professional staff (Conway and Dobson, 2003).

In relation to the careers of women in Australian regional universities, two studies examined the impact of Athena SWAN in a regional university. Nash et al. (2021) argued it could potentially be undermined by unintended reproduction of gender inequality in the academic workforce, while Manyweathers et al. (2020) observed that a key policy in the institution’s AS application providing support for care-givers travelling on university business was subject to gate-keeping, which meant they could not access the funds. Redmond et al. (2017) provided case studies of women leaders often from a rural background and the first in family to go to university. Regional location generated challenges for women in attending conferences or meetings within their discipline, and accessing mentoring and professional development (Wallace, 2005). Moreover, the need to travel could become a barrier to progression and career trajectories, as well as networking opportunities (Manyweathers et al., 2020). Although the use of video conferencing and Skype could overcome these barriers (Thomas et al., 2019; Herman and Hilliam, 2018, p. 186) a United Kingdom study found such technology isolated women on outlying campuses and reduced ‘their opportunities for career enhancing roles and access to informal and formal networks’. That institution therefore invested in new technology to enable remote participants to actively engage in large face to face meetings.

The current study will examine whether or not the terms of employment of women at an Australian regional university affected their working lives, their career aspiration, and career trajectories, and if barriers to career progression identified in this literature review impacted on them.

The theoretical framework is feminist institutionalism which acknowledges that gender exists in the practices, processes, ideologies and distribution of power in institutions (Acker, 1990) and provides a means of addressing the gendered nature of institutional change. Mackay et al. (2010, p. 580) argue that gender is an element constituting ‘social relations based upon perceived (socially constructed and culturally variable) differences between women and men, and as a primary way of signifying (and naturalising) relations of power and hierarchy’. Feminist institutionalism thus exposes how informal (gendered) interpretation of institutional rules impedes gender equality by highlighting ‘their informal and implicit nature’ (Clavero and Galligan, 2020, p. 662). It can therefore provide a framework to address the gendered nature of institutions and institutional change.

## Methodology

The principal researcher circulated an email via the university’s daily e-newsletter inviting all women staff to participate in a research project on the challenges for women working in Australian regional universities, which had approval from the university’s Human Research Ethics Committee. Potential participants were advised that if they agreed to take part in the project, they would be invited to give written responses to questions provided. Because there is a gap in this area of research, the researchers wanted to listen to women’s voices and provide an opportunity for them to be heard. They considered a qualitative methodology was more appropriate than a quantitative one in facilitating women’s narratives and their experiences being shared. To ensure women had the opportunity to tell their stories and to gather data from a wide range of sources, the researchers made a decision to work with every response received. The written narratives allowed women to tell their story in their own way so that it was agentic and personalized. It also meant they could answer the questions in their own time and as these women were often time poor and preferred flexible arrangements it seemed like this approach would be helpful for them. Such written accounts were an efficient means of gathering rich data and could provide more highly focussed and reflective data than oral interviews (Handy and Ross, 2005). They could also save the expense and time involved in face-to-face interviewing. While the relationship between the researcher and respondent may be more physically distant (Handy and Ross, 2005), the detail and quality of the information, described as “thick descriptions” (Miles and Huberman, 1994, p. 10), can enable the researcher to get to know the respondents.

The written responses were organised into categories that corresponded with the questions, and data was coded within these main categories, to form sub-categories (Kuckartz, 2019), and then analyzed. Direct quotes from these responses are listed by number; for example, Participant two appears as (P2). We gave a great deal of thought to preserving anonymity of participants. We did not collect data on age or the position which they held. Where appropriate in the results section we identify participants as either academic or professional staff and/or as managers. We have tried to provide context for quotes while at the same time preserving anonymity.

In Australian universities, professional staff are categorized as Higher Education Workers (HEW), beginning at Level 1. Staff members at the base of this level would not be required to have formal qualifications or work experience upon engagement. Higher levels would require post-secondary qualifications, while those working at the highest levels, Levels 9 and 10, would often have post-graduate qualifications. Academic staff at Level A are lecturers who work with support and direction from those at Lecturer level and above. Level B are lecturers, Level C senior lecturers, Level D associate professors and Level E full professors. Professional women working at HEW levels 9 and 10 would generally be managers and academic level E would often be heads of department or have a leadership role within their faculty.

This article focuses on responses to questions about employment which followed initial questions about participants’ identity–where they grew up, the languages they spoke and whether or not they identified as carers. In relation to employment, they were asked: What is your employment level? Eg. Academic A, B, C, D, E (full professor) or Higher Education Worker (HEW) level (for professional staff)? What is your employment status? - Sessional, part-time contract, full-time contract, part-time ongoing, full-time ongoing? For how many years have you worked as an academic/professional staff. In addition, a later question on the list asked: Do you feel the terms of your employment (fraction, employment/contract-type) have impacted on your career progression? The concept “terms of employment” therefore has three categories: full-time, part-time, and casual. But it has two dimensions, each with two categories: full-time/part-time (hours per week); and temporary/permanent (the former also known as casual or contract-based). It is estimated that in some Australian universities more than 80% of staff under the age of 30 are insecurely employed (Bone, 2019). Casual academic work can be a “double-edged sword” (Richardson et al., 2019). While some enjoy the flexibility of not having to attend meetings and annual performance reviews, they miss out on being part of an academic community which includes opportunities for conference travel, professional development and promotion. Much attention has been focused on academics as sessionals, but professional staff can also be casuals and only paid on an hourly basis. Casuals do not receive holiday or sick leave and can be unpaid during the midyear and summer breaks (Heffernan, 2019). An investigation found that 63% of workers at the eight largest universities in the state of Victoria in Australia were casual or on fixed-term contracts, and women accounted for 57% of these workers (Heffernan, 2019). In 2020 an Australian Senate parliamentary inquiry into wage theft of casual employees in universities called some universities to account for their employment practices. The enterprise bargaining agreements (EBAs) of universities – which regulate terms and conditions of employment and are negotiated by the union branch at each university and institution management and are voted on by employees - provide casuals with few employment rights and casual academics can effectively be paid by results rather than an hourly rate (Fenton and Kane, 2020); for example, there were claims that casuals were given only 10 min to mark each student’s examination paper (Maslen, 2020).

## Results

Ten of the twenty-one participants were professional staff working at HEW 2, 5, 6, 7, 8 or 9. Eleven were academics, half of them at Level B or lecturer level. The employment status of participants was mainly full time ongoing (13) with the remainder having full time fixed-term contracts, 0.8 contracts (that is; working 4 days a week), part-time contracts and two were sessionals (short fixed term contracts).

Years of service of participants in this study varied from a few months to 21 years. Mostly, those who had been at the university for more than 10 years had full-time ongoing employment, although one academic with 2 decades of employment was still on a fixed term contract. Several participants had started their careers as casuals/sessionals and progressed to contracts before securing ongoing positions. This mirrors findings on the growth of contract and casual positions in academia, often resulting in slower career progression for women (Strachan et al., 2016) who are more likely to be employed as sessional workers, at lower pay levels and have interrupted career development. They are therefore effectively held back in ways that men are not, often making tenure and progression elusive (Pyke 2013).

The responses of participants regarding how their employment affected careers varied. About half reported that it had no impact on them personally but some of these women acknowledging it had impacted on colleagues. Forty per cent of these were professional staff, and 60% were academic staff. The other half thought it had a significant effect on their careers. They also addressed how management responses to the terms of their employment affected career trajectories.

Ten participants in the current study said employment status had no impact or was not applicable to them; for example; one had full-time ongoing employment “so probably this has been positive” (Participant 1 (P1)). Another reported that it had no impact, adding that she “always had choices” and had adjusted working conditions to suit particular circumstances. The most difficult time had been returning part time after a year’s maternity leave “as I felt that I was not as connected to the workplace and the team, but this feeling has eased now that I have increased my days” (P2). While the following woman was clear that the terms of employment had not affected her personally, she alluded to the effect of broader organisational pressures; it was “more about the culture and work arrangements that have impacted’ (P8), and others complained about the lack of flexibility in working conditions (P9) and high workloads which delayed career progression (P17).

Even for this group, whose terms of employment had not affected career development, flexibility was key. One preferred short-term contracts, although she could see that “for a person with different personal circumstances, not knowing if your contract will get renewed it can be really stressful and a reason to look for a more secure job” (P7)’ Another had changed from full-time to part-time work to suit her particular circumstances (P14). In one work team, some members actually wished to work as sessionals to juggle various responsibilities, while others favoured fixed contracts:

have team members who prefer to be employed on rolling 13-weeks sessional contracts rather than fixed term arrangements as they believe this is more flexible for them in terms of managing other responsibilities outside of work. Others definitely prefer knowing that [their] employment is locked in for more significant periods (P16).

This comment suggested that women may make conscious choices about terms of employment to suit their particular needs. As Barrett and Barrett (2011, p. 152) observe: “career progression for women in HE is a stubborn, complex, equality issue”. Therefore, it was not surprising that the remaining 11 participants thought that their employment status had had an impact on their career progression or that of colleagues and their narratives often focused on the choices available to them. Their careers had not followed the typically male linear career model (White, 2014). They had often been pushed to the organisational fringe (White, 2013) where their contribution was not acknowledged and they did not feel part of or valued by the institution. Their status reflected what Crimmins (2016, p. 50) calls the “marginalised space” precarious academics feel they occupy. One woman described how she had moved from one short term contract to another:

I felt insecurely employed as a PT researcher on rolling 12-weeks contracts … In my current role the contract is longer and more substantial (12-months, full time) – I feel more securely employed now. I believe my career progression is partly hindered by these employment types, but perhaps more so by my levels of ambition, confidence etc. I feel capable of bigger things in one respect (I have the interest and ability) … but when it comes to practically navigating a more challenging career I don’t cope as well and don’t feel cut out for the uncertainty (P3).

This narrative suggests that career aspirations affect choices around flexible work and therefore terms of employment. Her career had been characterised by part-time, short-term contracts followed by her current 12-months full-time contract and consequently she felt “more securely employed now”, a common pattern in Australian universities (Strachan et al., 2016; Heffernan, 2019). It mirrors Crimmins (2016, p. 51), observation about “financial stress and anxiety” resulting from insecure jobs. This precarious employment had taken its toll on her wellbeing, reflecting Morrish (2019) findings; she did not “feel cut out for the uncertainty”. She tended to see her career progression being hindered by “my levels of ambition, confidence etc.” as well as short term contracts, but did not articulate a possible link between the two. Morley and Crossouard (2016, p. 164) describe this situation as misalignment where self-doubt, shame and humiliation, but also anger and resistance, risk the misaligned or ‘alien bodies’ simply ‘disappearing’ from view’. While she felt “capable of bigger things” she was exhausted by the challenge of navigating career progression, echoing the sentiments of women in White (2005), Kloot (2004) and Pyke’s (2013) studies.

This sense of being marginalised and disconnected from the institution by employment status was reflected in the narratives of several women at lower teaching levels, but other research suggests that even more senior women can be marginalised and see themselves as outsiders (Burkinshaw and White, 2017). Some women in this study were not permitted to fully participate in the university. One was a sessional academic who reported that she didn’t “feel part of the [university] community … would love to have an ongoing, permanent job” (P 5) while another sessional low-level academic was angry about the negative impact of her employment status on her career:

YES–Hell yes. I am not allowed to be on a committee and I can’t contribute to the university in that way. I cannot do the research that I want to because I have to work with someone else. As a sessional I cannot get ethics approval to be the principle (sic) researcher (P 6).

She considered that the university limited her participation in the broader academic community and saw sessionals as teachers, not researchers. Her perception of the unfairness of workload/task allocation, reflected the unfair treatment in Pyke’s 2013 study and also Barrett and Barrett (2011) observation that women were being railroaded in their career ambitions. All three of the previously mentioned participants were positioned as outsiders on the inside (Gherardi, 1995), that is, having a sense of not belonging in their workplace or, as Crimmins (2016, p. 51) notes of sessionals in her study: “the lack of fulfilment leaves them with a sense of exclusion or ‘outside-ness’”.

Regional location featured in discussion about choice and careers. Several participants commented that living and working in a regional location provided more flexibility. One mentioned “the opportunity to live and work in a regional location and to be able to work flexibly as needed when family and other circumstance require this” (P16). Two others (P1 and P2) said that the university being close to home was a benefit, and a third added: “local, not travelling to [other cities]” (P13).

However, one recognised that the regional location of the university and lack of mobility had affected her career.

I do believe that my lack of experience at other Universities has hindered my ability to progress or be considered for other roles, but staying in [a regional location] has been as a result of my family commitments … I have prioritised my family’s needs for a stable environment and therefore have not been able to get the broad experience that seems to be required to progress within the organisation (P4).

The choice to live in a regional area was guided by the need to provide a “stable environment” for her family. But it came at the cost of career progression in the university where she perceived that “broad experience” was required to change roles or move into more senior ones. This concurs with Wallace (2005) and Thomas et al. (2019) research at other Australian regional universities which concluded that regionality and the need to travel were additional complexities that could impact on other opportunities.

Four participants in the current study considered that the choices they made in order to balance work and care/household responsibilities had impacted on career progression. Lynch et al. (2012) argue that only women who divest themselves of care by not having children or if they have them waiting until they are grown up or ensuring they have a partner who supports them, will succeed in senior jobs in universities. Implicit in this view is that in the early career stage where the demands of family and work collide (Caprile, 2012), women will experience challenges in building careers.

One participant discussed the limited choice for job sharing or part-time roles in more senior positions.

Yes, when I first returned on a part-time basis due to my parenting and carer role, my opportunities to take on other roles were limited by my availability. Not many part-time roles are available at the higher … levels, nor job-sharing a common practice here (P19).

This narrative echoes Barrett and Barrett’s (2011, p. 153) reflection that while inevitably some women ‘will have slower progression in HE owing to personal choices that result in career breaks and/or a higher incidence of part-time working’; there was ‘a danger of this being exacerbated by inequitable treatment’. There is a tension here between the woman’s “choices” and perceptions that accommodating these would impact negatively on the organization. However, while job-sharing and part-time roles are often not available or encouraged at higher levels in universities (see White, 2017), they can have a positive impact on the organisation in terms of productivity and communication, and on the women job-sharing and their leadership development, as Watton et al. (2019) found.

Health issues as well as care commitments could also affect employment status, choice and career progression: “Yes definitely! I need the time to manage my health. … I also work part-time due to household responsibilities” (P 12). Another considered that her choice to take a gap in her career for carer responsibilities in an earlier job, had had “greater impact” “in that I have missed out on much further career progression that would have taken place during those gap” years (P15). This preference for part-time work, short-term contracts and taking time out of the workforce as a way of managing competing demands reflects the findings of Lewis et al. (2008, p. 25), that this was “recognisably *the* way of reconciling work and family”. Nevertheless. when a fractional appointment could be successfully negotiated it could be beneficial, as one participant noted: “so in fact it has benefitted me” (P 14). Another health issue was that the travel required between campuses could be logistically difficult, exhausting, and add to an already heavy workload; but this was not acknowledged by management: “Travel can be tiring; however, the University does not recognise the impact on time lost whilst traveling, excess hours worked and the work that needs to be made up due to the travel time” (P13). The implication here was that the university lacked any formal policies or acknowledgement of the time and impact of travel on employees. Travel between campuses was deemed to be a private matter that staff sorted out for themselves. The time taken to travel was not factored into the workload on their home campus. Other participants also mentioned that travel was tiring but necessary and impacted on their responsibilities as carers: “I find it more tiring than just working at my local campus BUT if it means more work (and more pay) then you just have to make the sacrifice” (P6), and “I get very tired driving … I find that I am less present for my family following travel due to fatigue”. (P4). There was no sense in these narratives that the requirement to travel could be negotiated with managers.

Several participants talked about their choice to work flexibly and less than full time, which can be contested territory in higher education. On the one hand, institutional gender equality policies support flexible work options for women (Fitzgerald and Wilkinson, 2010), but on the other, managers can be reluctant to support flexible work arrangements and can even question commitment to the job of women who seek such arrangements. This can lead to the derailing of careers for women with and even those without caring responsibilities (the potential of their maternal status can be ‘a hidden obstacle’) (Bevan and Gatrell 2017, p. 133). While one participant was able to purchase additional leave (known as 48/52; that is, purchasing an extra 4 weeks leave on top of the standard 4 weeks leave each year) (P 9), another remarked that her employment status had not affected her career because she had worked full-time in ongoing positions, but was aware that if she

… wanted to work at a reduced fraction it would be viewed negatively and seen as a lack of commitment to the organisation rather than a wish for better work-life balance. Whenever I advocate for my fractional staff or requests for workplace flexibility for my full-time staff (48/52 etc) I am made aware that it’s not the preference of my [manager] and have to fight to maintain fractional staff or implement flexible options (P11).

Here, a woman in a managerial role knew that the institution would not support her working less than full time and regarded any request to work a “reduced fraction” as a “lack of commitment to the organisation” and reinforced the sense that careers should follow a male linear model. These comments reflect the previously expressed views of Participant 19 about the lack of part-time roles at higher levels and corresponds with White’s (2017) observation that if senior women request part-time work, this can be seen as demonstrating less commitment to the organisation and thus a career limiting move. Another participant reflected on how the inability to travel could leave women conflicted between the desire to undertake training as expected by managers and family demands:

This is highly difficult. I understand it is a part of the role. However, with limited options, sometimes this can be very conflicting. I often miss opportunities. Often, I would like to attend but am unable to–or I am unable to justify the upheaval required for my own wishes. This is conflicting for the role/uni and for my family. This is often a no-win situation, feeling either burdensome and guilty or unreliable/uncommitted (P9).

Disturbingly, these narratives suggest that choices can be limited and some senior managers did not endorse flexible work options despite university policy promoting such flexibility. It was not clear if they opposed these options or if they labelled women who took up flexible options as ultimately privileging the domestic sphere above the professional career domain. Moreover, it indicates the challenges for line managers who try to advocate for fractional staff or flexible work options for themselves and/or their full-time staff; and concurs with Bailey et al., 2017 findings that few senior women professional staff in HE worked part time. In addition, the travel required between campuses was not recognised by management as work time and women with children were not necessarily supported to undertake training away from their campus. While there are questions about how the choice to work flexibly could lead to different and lesser career paths (Padevic et al., 2019), some women in this study were not given a choice about working part time or flexibility within full-time positions, despite such choices being enshrined in federal legislation. Whether this was the result of informal expectations of managers that committed workers would be in full-time and fixed positions or whether these options were not apparent in policy and practice in the workplace, the data clearly indicated that flexible work options were not always supported by management.

**Table:** 

Theme 1	insecure employment of some participants had an impact on wellbeing and confidence
Theme 2	care/household responsibilities had a negative effect on career progression
Theme 3	the choice to work flexibly impacted on careers
Theme 4	managers did not necessarily support flexible work options such as working part time or reduced hours or job sharing, and they did not support women who had to juggle travel between campuses with high workloads/care responsibilities

## Discussion and Conclusion

This study examined how women working at an Australian regional university perceived that the terms of their employment had impacted on career progression. While ten women reported it had no impact on their careers, some were aware of the influence on colleagues who preferred to work as casuals or to have ongoing employment. Nevertheless, this group identified employment issues such as difficulties when returning from maternity leave, lack of flexibility in working arrangements, the broader institutional culture, and high workloads, also noted in other research (see for example Burkinshaw and White, 2017). But half the participants did consider that the terms of employment impacted on career progression.

Four main themes emerging from the data were: insecure employment and the impact on wellbeing and confidence; the effect of care/household responsibilities on career progression; the impact of the choice to work flexibly; and managers not necessarily supporting flexible work options.

Insecure employment for women in Australian HE has been widely recognised (for example, Strachan et al., 2012; Strachan et al., 2016), especially for those under the age of 30 (Bone, 2019). Insecurity for participants was sometimes exacerbated by living in a regional location with limited alternative job options. Moreover, some women in this study who were casuals emphasized their precarious employment and did not feel that they were part of the university; they had a sense of not being connected or valued, which concurs with Francis and Stulz (2020) research. Their difficulty in settling into their role led to uncertainty and stress. They could not sit on university committees or submit ethics applications to undertake research projects, and perceived that they were unable to influence institutional policy or effectively be part of the organisation; they were essentially outsiders on the inside (Gherardi, 1995; White, 2013). There appeared to be a link between casual employment and confidence in their abilities, which tended to keep women at the margins (Bevan and Gattrell, 2017) rather than believing they could advance in their careers (Burkinshaw and White, 2017). Being on the outside or what Crimmins (2016, p. 53) describes as “otherness” suggested a two-tier employment model where sessionals/casuals in this study were not appreciated by management and were exhausted by the uncertainty of insecure employment, like academics in Crimmins (2016), p. 53 research who felt marginalised “on the periphery of academia, with little opportunity of becoming an ongoing academic or informing policy”.

The second theme was the effect of care/household responsibilities on career advancement. Those women who decide to combine motherhood with a career can experience a maternal wall (Williams, 2004); that is, colleagues can view mothers or pregnant women as less competent or less committed to their jobs. Thus mothers (and some fathers) can be penalised in their careers (White, 2014). As Bevan and Gatrell (2017, p. 180) observe, women’s motherhood or even potential for motherhood can place them “on the sidelines … as they struggle both with the practical challenges of managing a home and career as well as experiencing unfair assumptions about their career orientation”. Some women in the present study had prioritised family over career, had tried to negotiate part-time roles, had taken time out of the workforce, and had requested job sharing. Their experience reflected Bailey et al., 2017 findings that part-time work, which can impede career advancement, was used extensively by lower classified professional women in Australian universities, but rarely by higher classified women. These women talked about missing out on career advancement because they had prioritised caring over careers. Some also expressed a strong preference to live in a regional location as compatible with their sense of belonging (Goriss-Hunter and Burke, 2015) and family responsibilities, even though this might reduce career mobility, networking and development opportunities, as Thomas et al. (2019) also found.

The third theme, the impact of choosing flexible work options, was often negative. While there was evidence that certain women chose to work as casuals or sessionals as a way of managing work and other responsibilities, it was a double-edged sword (Richardson et al., 2019). Strachan et al. (2016) and Crimmins (2016) would argue that sessional workers in HE were not sessional by choice, although some might choose casual employment as an entry level position into academia but then end up being trapped. Other women in this study purchased extra leave, which gave them 8 weeks leave per year. But even this type of limited flexibility might not be supported by managers. Flexibility then, could be a career-limiting strategy and place women on a different, and lesser, career trajectory to other employees in the organisation (Barrett and Barrett, 2011; Padevic et al., 2019).

The fourth theme demonstrated how informal, gendered rules can predominate in universities. Managers did not readily support flexible work options, despite institutional and national imperatives, supporting instead a conventional linear male career model. There was evidence that some senior managers preferred staff to be in full-time roles, to juggle travel between campuses with high workloads/carer responsibilities and did not support line managers negotiating flexible work for themselves or for their fractional or full-time staff, as also noted at another Australian university (White, 2017). The experience of several women in this study mirrors Tutchell and Edmonds (2015, p. 216) observation that women often considered they could not take the necessary breaks “without damaging their jobs and promotional prospects” and being confident that a career with “the pauses, the deviations and the changes from full time to part time and back again that women need, is valued as highly as the linear uninterrupted career conventionally worked by men”.

Even greater strain will be placed on university employment and budgets by COVID-19 (Blackmore, 2020). Women are shouldering more of the caring responsibilities and could be at greater financial disadvantage, particularly during COVID-19 lockdowns with remote schooling, domestic chores and working from home (WGEA, 2020). The impact is that women, especially those in regional areas with a lack of alternative employment, are likely to experience even more insecure employment and pressure in their jobs (Ferrari, 2020).

Feminist institutionalism has been a useful framework for this study. It has enabled us to understand why the participants experienced difficulties with career progression. While the university in this study, like all Australian universities, has policies and strategies to achieve gender equality for staff, the informal practices or what Clavero and Galligan (2020, p. 662) call “informal (gendered) institutional rules” demonstrated an active institutional resistance to gender equality, including little support for women and men who wished to explore more flexible work options that better suited their need to balance work and other responsibilities (Meekes, 2021). Several of the women were employed either part time or as sessionals and their working conditions assigned them to the margins of the university, as though gender equality policies did not apply to them which begs the question of how effective gender equality policies in Australian higher education have been (Diezmann and Grieshaber, 2019). Others considered they were penalised for requesting fractional appointments or job sharing that might make it easier to balance work and caring responsibilities, despite national legislation giving them the right to negotiate these arrangements. The most overt examples of informal and gendered institutional rules that discriminated against women were senior managers favouring a linear uninterrupted male career model by refusing requests from line managers for their female staff to have various flexible work options, and no recognition of how travel between campuses impacted on workloads and carer responsibilities. These practices demonstrate that the masculinist status quo in universities is resistant to change (Ash, 2021). Until managers seriously address gender equality in higher education institutions and it becomes embedded in workplace culture (Wroblewski, 2017), women’s career progression will continue to be precarious.

In conclusion, this study of women working at an Australian regional university examined the link between levels of employment and career progression. It found that their employment was possibly more precarious than for women at metropolitan universities, because of limited alternative job options. Moreover, some senior managers endorsed a linear male career model and did not support requests for flexible work options, while also marginalising casual (sessional) employees, and not acknowledging the impact of travel between campuses on workloads. Hence women could be dissuaded from requesting fractional appointments or job sharing that might enable them to combine work and caring responsibilities. These informal practices of some managers were inconsistent with both the university’s obligations under national legislation that enable employees with responsibility for the care of a child to negotiate flexible work arrangements, and implementation of its institutional gender equality policies.

## Data Availability

The datasets presented in this article are not readily available because the data set has been anonymised and is held in a secure location by the researchers. Requests to access the datasets should be directed to kate white kate.white@federation.edu.au and anitra goriss-hunter, a.goriss-hunter@federation.edu.au.
